# Effects Of treadmill training on hindlimb muscles of spinal cord–injured mice

**DOI:** 10.1002/mus.25211

**Published:** 2016-11-07

**Authors:** Camila R. Battistuzzo, Michelle M. Rank, Jamie R. Flynn, David L. Morgan, Robin Callister, Robert J. Callister, Mary P. Galea

**Affiliations:** ^1^ Department of Medicine (Royal Melbourne Hospital)The University of MelbourneParkvilleVictoria3010Australia; ^2^ School of Medical SciencesRMIT UniversityMelbourneVictoriaAustralia; ^3^ Faculty of Health and Medicine, School of Biomedical Sciences and PharmacyThe University of NewcastleNewcastleNew South WalesAustralia

**Keywords:** hemisection, mice, muscle atrophy, muscle fiber type, spinal cord injury, treadmill

## Abstract

Introduction: Treadmill training is known to prevent muscle atrophy after spinal cord injury (SCI), but the training duration required to optimize recovery has not been investigated. *Methods:* Hemisected mice were randomized to 3, 6, or 9 weeks of training or no training. Muscle fiber type composition and fiber cross‐sectional area (CSA) of medial gastrocnemius (MG), soleus (SOL), and tibialis anterior (TA) were assessed using ATPase histochemistry. *Results:* Muscle fiber type composition of SCI animals did not change with training. However, 9 weeks of training increased the CSA of type IIB and IIX fibers in TA and MG muscles. *Conclusions:* Nine weeks of training after incomplete SCI was effective in preventing atrophy of fast‐twitch muscles, but there were limited effects on slow‐twitch muscles and muscle fiber type composition. These data provide important evidence of the benefits of exercising paralyzed limbs after SCI. *Muscle Nerve*, 2016 *Muscle Nerve* **55**: 232–242, 2017

**Abbreviations**
ANOVA: analysis of variance
ATP: adenosine triphosphate
CSA: cross‐sectional area
MANOVA: multivariate analysis of variance
MG: medial gastrocnemius
SCI: spinal cord injury
SOL: soleus
TA: tibialis anterior

Spinal cord injury (SCI) leads to significant changes in muscles innervated below the level of injury.^1^ After SCI, muscle atrophy, altered contractile properties, and muscle fiber type transformations have been observed as early as 4–7 days after injury.^2^, ^3^, ^4^, ^5^ These changes, and the extent of muscle recovery, are dependent on the severity of the injury.^1^, ^6^ Although muscle properties have been extensively investigated in models of complete SCI,^2^, ^5^, ^7^, ^8^ the pattern of muscle atrophy and recovery after incomplete SCI, which imposes different stresses in the muscles, is less clear.^1^

One way of reducing muscle atrophy after SCI is to exercise muscles with innervation below the lesion via treadmill training. This type of intervention has been shown to prevent some of the detrimental muscle changes that occur after SCI.^9^, ^10^ For example, 1 week of treadmill training leads to an increase in soleus muscle cross‐sectional area in rats with SCI.^11^, ^12^, ^13^ Despite positive evidence of that treadmill training prevents muscle atrophy, most studies have been of short duration (5–7 days) and have only assessed the effect of treadmill training in 1 or 2 muscles. As each muscle may respond in a different manner, investigating the effect of training in a variety of muscles is important if we are to improve activity‐based therapies for patients with SCI. Thus, the aim of this study was to investigate the effect of incomplete SCI and different durations of treadmill training (3, 6, and 9 weeks post‐injury) on hindlimb muscle properties in mice. Both the affected (left) and non‐affected (right) limbs were examined to determine injury‐specific training effects. An analysis of hindlimb muscle properties was also conducted in uninjured (control) mice with and without treadmill training to control for the effect of age.

We hypothesized that SCI mice that undergo 9 weeks of treadmill training would have less muscle atrophy compared with 9 weeks untrained mice or mice undergoing 3 or 6 weeks of training.

## METHODS

### Experimental Design

**Figure 1:**
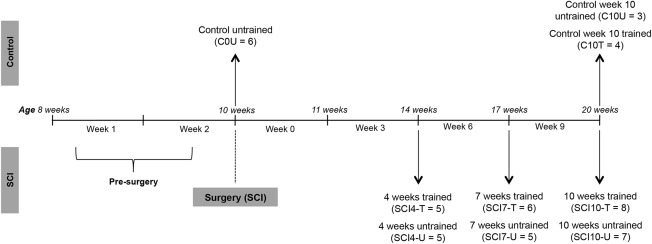
Experimental design timeline. SCI, spinal cord injury.

A total of 49 male C57BL/6 mice were used in this study. SCI animals (age 10 weeks at surgery, body weight ∼20 g) were randomized to treadmill trained or untrained groups. Animals allocated to the treadmill‐trained groups received daily treadmill training for 3, 6, or 9 weeks and were then euthanized at 4, 7, and 10 weeks post‐injury, respectively. Control animals (age‐matched without SCI) were used to compare the effects of aging over the maximum time frame of the SCI experiments. One control group was euthanized at the time of surgery in SCI animals. Two other control groups were randomized to either 9 weeks of treadmill training or no training and were euthanized at the end of 10 weeks (Fig. 1). The designations used to describe the characteristics of the groups are shown in Table 1. Examples of the group designations are C10U (control, 10 weeks, untrained) and SCI4‐T‐N (SCI, 4 weeks, trained, non‐affected limb).

**Table 1:** Group designations for comparisons

Variable	Categories
Injury status	Control (C), spinal cord injury (SCI)
Time (weeks post‐injury)	0, 4, 7, 10
Training status	Untrained (U), trained (T)
Limb status	Non‐affected (N), affected (A)

### Animal Care and Surgical Procedures

All procedures were approved by the animal care and ethics committee of the University of Newcastle. Animals were maintained on a 12‐hour light/dark cycle with laboratory rodent chow and water *ad libitum*. A maximum of 4 animals were housed per cage, regardless of their treatment allocation.

All animals in the SCI group received a lateral spinal cord hemisection at the level of the T10 vertebra, as described in an earlier study.^14^ Briefly, mice were anesthetized with isoflurane (5% induction, 1.5%–2.5% maintenance) and medetomidine (0.03 mg/kg subcutaneously). The vertebral column was then exposed by a midline incision over T7 to L1, and an ophthalmic knife was used to create a left hemisection at T10 (i.e., between T10 and T11 spinal roots). The overlying muscle was then sutured, and the skin incision was sealed using 7‐mm staples. All procedures were conducted under aseptic conditions. Post‐surgical analgesia was provided with buprenorphine (0.1 mg/kg subcutaneously, every 8 hours for 48 hours) and carprofen (5 mg/kg subcutaneously, every 24 hours for 5 days). Animals were observed closely for the next 48 hours for signs of pain or distress. The surgeon was blinded to experimental group allocation.

### Treadmill Training

Two weeks before surgery, all animals were familiarized with treadmill training at approximately 10 m/min for 10 min, twice per day, 5 days per week (6‐lane treadmill; Simple II; Columbus Instruments, Columbus, Ohio). One week after SCI, animals were randomly assigned to treadmill‐trained or untrained groups. The trained group then recommenced the training regimen as before injury and continued for 3, 6, or 9 weeks. After injury, treadmill speed was initially 6 m/min and was gradually increased to 10–12 m/min over the course of training. Gentle tapping of the tail or hindlimb was used to encourage running if the animal stopped. There was no significant difference in body weight between trained and untrained animals at any time‐point.

### Muscle Histochemistry

Animals were euthanized by decapitation under deep anesthesia (ketamine 100 mg/kg intraperitoneally). The medial gastrocnemius (MG), soleus (SOL), and tibialis anterior (TA) muscles were then dissected from the right (non‐affected side in SCI) and left (affected side in SCI) hindlimb of all animals, including controls. These muscles are important dorsiflexors (TA) and plantarflexors (MG and SOL) of the ankle joint. The muscles were frozen in isopentane and stored at –80°C. Serial cross‐sections (10 μm thick) from the muscle midbelly were cut on a cryostat (Model 2800, Frigocut; Leica Instruments, Wetzlar, Germany) at −20°C and mounted on 22 × 22‐mm coverslips. Subsequently, ATPase histochemistry was applied according to the method of Callister *et al*. to determine muscle fiber type.^15^ Acid‐stable myosin adenosine triphosphatase (ATPase) activity was determined by pre‐incubating sections for 5 min in acetate/barbital buffer (0.1 M) adjusted from pH 4.2 to 4.6 (intervals of 0.1 pH unit). Sections were then rinsed 3 times with distilled water and incubated for 30 min in a solution containing 3.6 mM ATP, 18 mM CaCl_2_, and 20 mM Na barbital (pH 9.4). After this incubation, slides were washed twice in 1% CaCl_2_, immersed for 5 min in 2% CoCl_2_, washed 3 times in 10 mM Na barbital rinse, and developed for 1 min in 1% ammonium sulfide solution. Finally, slides were washed several times in running tap water, dehydrated in an ascending series of alcohols, cleared with xylene, and mounted on slides with Permount (Fisher Scientific, Pittsburgh, Pennsylvania).

### Muscle Measurements

**Figure 2:**
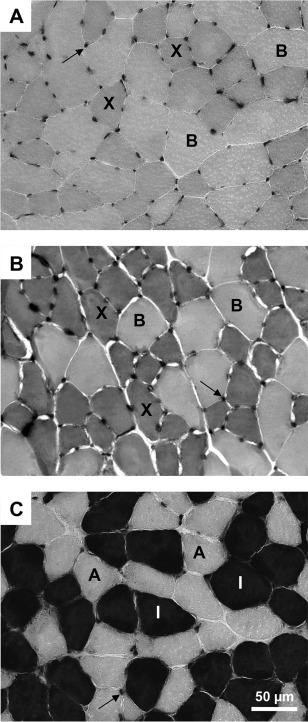
Serial sections of muscles from control untrained animals (C0U) stained for myosin ATPase activity. **(A)** Tibialis anterior muscle is composed of type IIB and IIX fibers (pH 4.4). **(B)** The deep part of the medial gastrocnemius muscle is composed of type IIB and IIX fibers (pH 4.3). **(C)** Soleus muscle is composed of type IIA and I fibers (pH 4.3). Staining intensity (dark to light) is: I > IIX > IIB > IIA. Arrows indicate capillaries.

Fields within stained cross‐sections were captured at 40 × magnification using a microscope with a digital camera (BX60; Olympus Optical, Ltd., Tokyo, Japan). A total of 4 sample fields were selected from all stained sections in each muscle to account for non‐uniform fiber type distribution. For example, in muscles with significant fiber type compartmentalization (such as MG and TA), the deep and middle parts were randomly sampled. Muscle fiber type and muscle fiber cross‐sectional area (CSA) were measured in approximately 200 muscle fibers from each muscle in each animal. The same fibers selected for CSA measurement were used to determine fiber type proportion in each muscle. Muscle fiber type was distinguished according to the staining intensity from the histological procedure described above (I > IIX > IIB > IIA, dark to light; Fig. 2). A blinded assessor performed all the muscle measurements manually using the AnalySIS Five software (Olympus Soft Imaging Solutions GmbH, Hamburg, Germany).

### Lesion Extent Measurements

Lesion extent measurements were described in our recent publication,^16^ which includes the same animals used in this study. Briefly, the spinal cord was removed and sliced horizontally for electrophysiological recording. The extent of the hemisection was then measured on images of the horizontal slices. The results of the lesion extent (distance between the medial apex of the lesion and the midline of the spinal cord) for the subset of animals included in this study showed no difference between untrained and trained animals (0.31 ± 0.01 mm^2^ and 0.32 ± 0.01 mm^2^, respectively; *P* = 0.9). The lesion area (cavitation and missing tissue) was also similar between the 2 groups (0.34 ± 0.02 mm^2^ and 0.32 ± 0.02 mm^2^, respectively; *P* = 0.5). These data indicate that the hemisection lesion was similar between trained and untrained animals.

### Statistical Analyses

The overall effects of SCI, limb side, and treadmill training were tested using multivariate analysis of variance (MANOVA) for all muscle fiber types. ANOVA with Scheffé *post‐hoc* analysis was then used to test for group differences. The same approach was used to analyze the effect of age (control animals). Statistical analyses were performed using Data Desk (version 6.3) software (Data Description, Inc., Ithaca, New York). All data are reported as mean ± SEM. Significance is reported as *P* < 0.05 or as indicated.

## RESULTS

### Fiber Type Composition in Control Animals

There was no statistically significant difference between the muscle fiber type composition of the right and left hindlimb muscles in control animals (*P* = 0.42). Therefore, data from both limbs from control animals were combined for subsequent analysis.

Both TA and MG muscles of control animals were predominantly composed of type IIB fibers, with a slightly higher proportion of type IIB fibers in MG compared with TA. In SOL, type IIA fibers predominated over type I fibers. There were no significant changes with age or training status for TA, MG, or SOL (Table 2).

**Table 2:** Effects of age and treadmill training on muscle fiber type composition in control mice

Muscle	Fiber type (%)	C0U	C10U	C10T
TA	IIB / IIX	72 ± 3 / 28 ± 3	69 ± 3 / 31 ± 3	67 ± 4 / 33 ± 4
MG	IIB / IIX	76 ± 4 / 24 ± 4	81 ± 2 / 19 ± 2	74 ± 2 / 26 ± 2
SOL	IIA / I	66 ± 1 / 34 ± 1	60 ± 2/ 40 ± 2	64 ± 3 / 36 ± 3

No statistically significant effect of age or training was found in any of the examined muscles of control animals. Results are expressed as mean ± SEM. TA, tibialis anterior; MG, medial gastrocnemius; SOL, soleus; C, control; U, untrained; T, trained.

### Fiber Type Composition in SCI Animals

**Figure 3:**
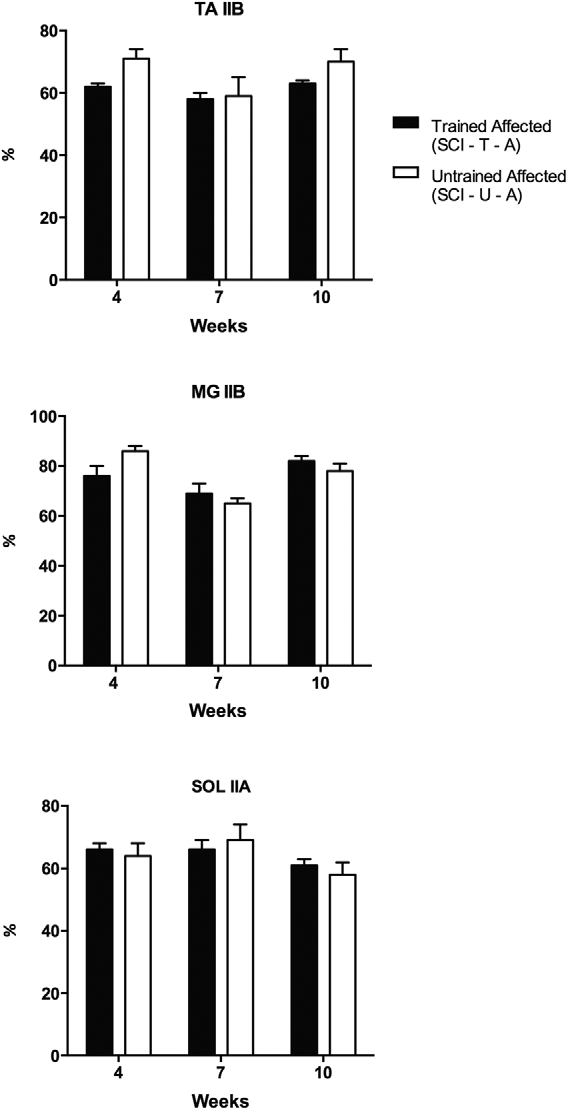
Muscle fiber type composition of affected hindlimb muscles in trained and untrained SCI mice at 4, 7, and 10 weeks post‐SCI. No significant difference was found between the muscle fiber composition from SCI‐T‐A and SCI‐U‐A animals at any time‐point. Results are expressed as mean ± SEM. TA, tibialis anterior; MG, medial gastrocnemius; SOL, soleus; SCI, spinal cord injury; U, untrained; T, trained; A, affected hindlimb.

To investigate whether treadmill training affects muscle fiber type composition in SCI mice, the predominant fiber types of affected hindlimb muscles of trained and untrained SCI animals were compared (Fig. 3). There was no significant difference between the 2 groups at any assessment in all 3 muscles. This shows that treadmill training had little effect on fiber type composition in the examined muscles.

**Figure 4:**
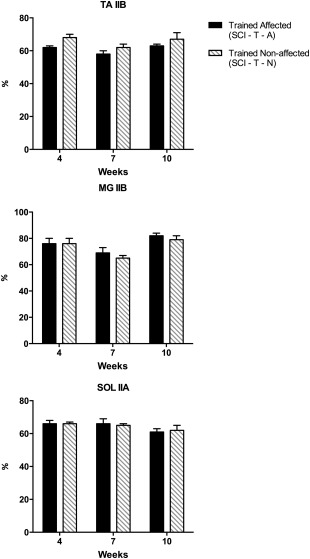
Muscle fiber type composition of the affected hindlimb muscles in trained and untrained SCI mice at 4, 7, and 10 weeks post‐SCI. No statistically significant difference was found in any of the examined muscles of control animals. Results are expressed as mean ± SEM. TA, tibialis anterior; MG, medial gastrocnemius; SOL, soleus; SCI, spinal cord injury; U, untrained; T, trained; N, non‐affected hindlimb; A, affected hindlimb.

The results from the comparisons of muscle fiber type composition between the affected and non‐affected sides (internal control) of trained animals also did not show a statistically significant difference in any muscle (Fig. 4).

### Fiber Type Composition in SCI Animals vs. Control Animals

To determine the extent of muscle fiber type composition change after SCI, muscles of the affected hindlimb of 9‐week trained and comparable untrained animals (SCI10‐T‐A and SCI10‐U‐A) were compared with the muscles from 10‐week untrained control animals (C10U) (Table 3). Overall, there were no significant differences (by ANOVA with Scheffé *post‐hoc* analysis) in muscle fiber type composition in the 3 muscles between SCI10‐U‐A and C10U animals (TA, *P* = 0.8; MG and SOL, *P* = 0.4). Similarly, 9 weeks of treadmill training (SCI10‐T‐A) did not significantly change muscle fiber type composition in SCI animals when compared with C10U animals (TA, *P* = 0.09; MG, *P* = 0.6; SOL, *P* = 0.7).

**Table 3:** Comparisons between muscle fiber type composition from the affected side of SCI and control animals at 10 weeks

Muscle	Fiber type (%)	C10U	SCI10‐U‐A	SCI10‐T‐A
TA	IIB	69 ± 3	70 ± 4	63 ± 4
MG	IIB	81 ± 2	77 ± 3	82 ± 2
SOL	IIA	60 ± 2	58 ± 2	61 ± 3

No statistically significant difference was found between SCI and control untrained animals at 10 weeks in any of the muscles examined. Results are expressed as mean ± SEM. SCI, spinal cord injury; TA, tibialis anterior; MG, medial gastrocnemius; SOL, soleus; C, control; U, untrained; T, trained; A, affected hindlimb.

### Muscle Fiber CSA in Control Animals

**Figure 5:**
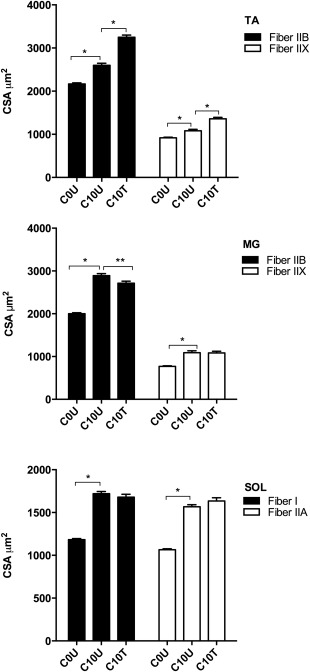
Muscle fiber cross‐sectional area (CSA) in control mice. The CSA of all fiber types examined in the 3 muscles increased significantly with age. Nine weeks of treadmill training influenced the CSA of TA (IIB and IIX) and MG (IIB) fiber types. Results are expressed as mean ± SEM. *P < 0.0001, **P < 0.001, and ***P = 0.002. C, control; 0 or 10, number of weeks in experiment; U, untrained; T, trained; TA, tibialis anterior; MG, medial gastrocnemius; SOL, soleus.

The CSA of all fiber types examined in the 3 muscles increased significantly with age (Fig. 5). The CSA in C10U was significantly larger than CSA in C0U for both fiber types in all 3 muscles (all *P* < 0.0001). The magnitudes of these CSA increases in C10U compared with C0U were 16% (IIB) and 15% (IIX) for TA, 31% (IIB) and 29% (IIX) for MG, and 31% (I and IIA) for SOL. There were also significant differences in CSA between C10U and C10T in both fibers of TA, with CSA in C10T greater (*P* < 0.0001) than that of C0U for both IIB and IIX fibers. The CSA of IIB fibers in MG, however, was significantly (*P* = 0.002) smaller in C10T than C10U; there were no differences between C10U and C10T CSA for IIX in MG or either fiber type in SOL.

### Muscle Fiber CSA in SCI Animals

**Figure 6:**
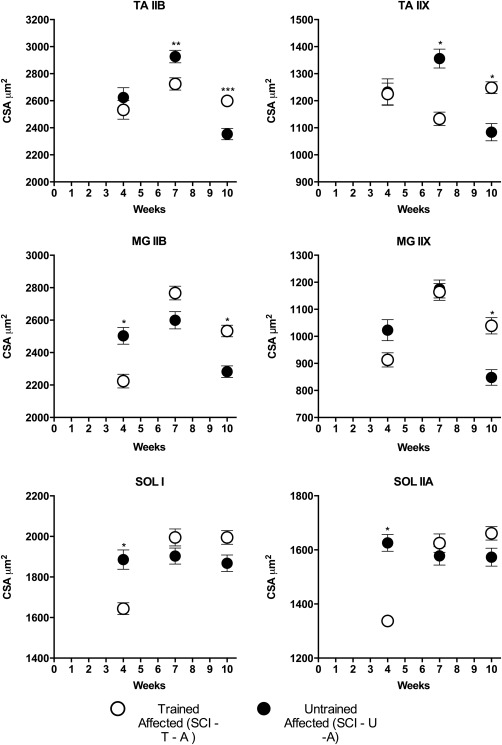
Muscle fiber cross‐sectional area (CSA) of the affected hindlimb muscles in untrained and trained SCI mice at 4, 7, and 10 weeks post‐SCI. At 10 weeks post‐injury, the CSA from TA muscle fibers of untrained animals was significantly larger compared with trained animals. No significant difference was found for MG SOL muscle fibers at this time‐point. Results are expressed as mean ± SEM. *P < 0.0001, **P < 0.01, and ***P < 0.001. TA, tibialis anterior; MG, medial gastrocnemius; SOL, soleus; SCI, spinal cord injury; U, untrained; T, trained; A, affected hindlimb.

The effects of training on the CSA of muscles of the affected hindlimb varied with the duration of training period (Fig. 6). For TA, there were no significant differences at 4 weeks, but both fiber types were significantly larger in untrained animals than in trained animals at 7 weeks (type IIB, 7%; type IIX, 16%), whereas, by week 10 post‐injury, the CSA of both IIB and IIX fibers was larger in the trained animals compared with untrained animals (9%, *P* < 0.001, and 13%, *P* < 0.0001, respectively). For MG, fiber CSA from untrained mice was significantly larger (11%) than that of trained mice (*P* < 0.0001) in IIB fibers. A similar trend was observed for IIX fibers at 4 weeks, whereas by week 10 post‐injury, the CSA of both IIB and IIX fibers from SCI10‐T‐A animals was significantly greater (10% and 18%, respectively) compared with SCI10‐U‐A animals (*P* < 0.0001). For SOL muscle, both type I and type IIA fiber CSA were significantly larger in SCI4‐U‐A than SCI4‐T‐A mice (*P* < 0.0001). No significant differences were observed at 7 or 10 weeks, although there was a trend for CSA in trained SCI10‐A to be larger than in untrained SCI10‐A at 10 weeks. In summary, muscle fiber CSA tended to be smaller in treadmill‐trained than untrained animals early in recovery but greater in trained than untrained animals by 10 weeks.

**Figure 7:**
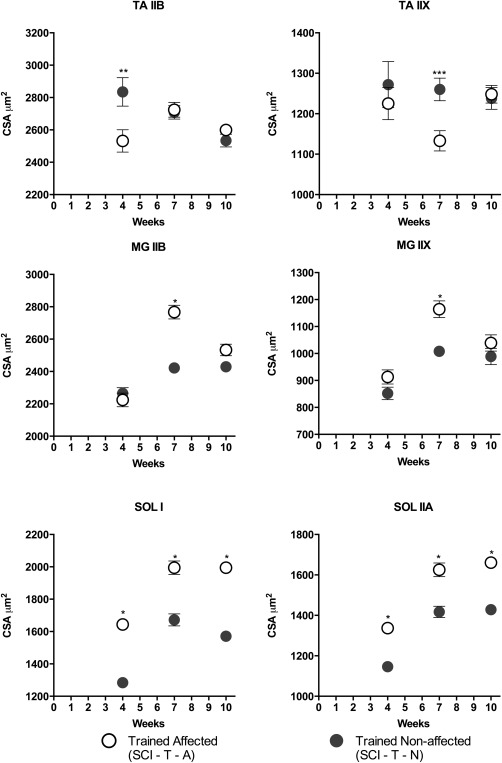
Comparison of the muscle fiber cross‐sectional area (CSA) of the non‐affected and affected hindlimb muscles in trained SCI mice at 4, 7, and 10 weeks post‐SCI. The CSA of both fiber types of the SOL muscle from the affected side were significantly larger compared with the non‐affected SOL at all time‐points. Results are expressed as mean ± SEM. *P < 0.0001, **P = 0.02, and ***P < 0.01. TA, tibialis anterior; MG, medial gastrocnemius; SOL, soleus; SCI, spinal cord injury; T, trained; N, non‐affected hindlimb; A, affected hindlimb.

Fiber CSA from the affected and non‐affected hindlimbs of trained SCI animals differed with the training period, muscle groups, and fiber types examined (Fig. 7). For TA, CSA from the non‐affected muscle was significantly larger (11%) than than that of the affected side at 4 weeks for IIB fibers (SCI4‐T‐N vs. SCI4‐T‐A, *P* = 0.002) and at 7 weeks for type IIX fibers (*P* < 0.01), but there were no differences by 10 weeks. For MG, CSA of the hindlimb muscle fibers was not different at 4 weeks, was greater in the affected‐side fibers at 7 weeks (*P* < 0.0001), but was no longer different at 10 weeks. For SOL, CSA was significantly greater in affected than non‐affected muscles in both fiber types at 4, 7, and 10 weeks (*P* < 0.0001).

### Muscle Fiber CSA in SCI Animals vs. Control Animals

The effects of muscle recovery after SCI and treadmill training on that recovery were examined by comparing the CSA of muscles on the affected side of untrained and trained animals (SCI10‐U‐A and SCI10‐T‐A, respectively) with the CSA of muscles from untrained control animals at 10 weeks (Table 4). The CSA of untrained SCI10‐U‐A was significantly less than that of controls (C10U) for TA IIB and MG IIB and IIX fibers. It was significantly greater than that of controls in SOL type I fibers, but not different in TA IIX or SOL IIA fibers. Treadmill training effects were the elimination (TA IIB) or decrease (MG IIB and IIX) in the magnitude of reductions in CSA between SCI10‐T‐A and controls, or increase in CSA in SOL (I and IIA) of SCI10‐T‐A compared with controls. This suggests that treadmill training was protective against muscle atrophy (MG) and selectively led to muscle hypertrophy (SOL) after SCI.

**Table 4:** Comparisons between muscle fiber CSA from the affected side of SCI and control animals at 10 weeks

					Comparison	
Muscle	Fiber type (μm^2^)	C10U	SCI10‐U‐A	SCI10‐T‐A	SCI10‐U‐A vs. C10U	SCI10‐T‐A vs. C10U
TA	IIB	2,598 ± 49	2,354 ± 42	2,599 ± 33	9%^a^↓	NS
	IIX	1,082 ± 32	1,084 ± 32	1,248 ± 22	NS	14%*↑
MG	IIB	2,892 ± 47	2,282 ± 37	2,533 ± 36	21%*↓	12%*↓
	IIX	1,090 ± 45	848 ± 29	1,039 ± 30	22%*↓	5%^a^↓
SOL	I	1,720 ± 26	1,868 ± 41	1,995 ± 35	8%^b^↑	13%*↑
	IIA	1,568 ± 23	1,574 ± 33	1,661 ± 25	NS	6%^c^↑

Arrows indicate whether the muscle cross‐sectional area (CSA) from SCI animals increased, decreased, or did not change significantly (NS) in relation to the muscle CSA of control animals. Results are expressed as mean ± SEM. TA, tibialis anterior; MG, medial gastrocnemius; SOL, soleus; C, control; SCI, spinal cord injury; U, untrained; T, trained; A, affected side.

a *P* < 0.0001;

b *P* = 0.02;

c *P* = 0.005.

## DISCUSSION

In this study we have systematically investigated the effects of different training durations on muscle fiber type composition and fiber CSA of incomplete SCI animals, as well as controlling for the effects of age and exercise training in uninjured control animals. The main findings of this study are that fiber type composition of the 3 muscles examined (TA, MG, and SOL) did not change with age in control animals, did not change in the affected or non‐affected limb after SCI, and was not influenced by treadmill training. Age and treadmill training influenced muscle fiber CSA in control animals after 10 weeks, and therefore consideration of these effects in this study were important for interpreting the changes that occurred after SCI. Muscle fiber CSA in the untrained affected limb of SCI animals increased initially and then decreased by 10 weeks in both TA and MG, but did not change in SOL. Treadmill training exerted benefits in stimulating fiber CSA, especially at 10 weeks, and were most pronounced in MG and least in SOL. Treadmill training effects on muscle fiber CSA were more evident in the affected compared with the non‐affected muscles of SCI animals. Together these findings support the therapeutic use of treadmill training and muscle loading after incomplete SCI.

Few studies have assessed the effects of long periods (i.e., months) of locomotor training on muscle properties after incomplete SCI, with the majority of studies using only 1 week of training.^11^, ^12^, ^13^ These short‐term studies showed that muscles more affected initially by SCI have a greater capacity for recovery after locomotor training than less affected muscles, which is consistent with our findings.^11^, ^13^ We have found only 1 study that has assessed the effects of long‐term treadmill training (i.e., up to 12 weeks) on hindlimb muscle properties after incomplete SCI. Liu *et al*. used MRI to measure whole muscle CSA in 4 hindlimb muscle groups of spinal contusion in rats after treadmill training for up to 12 weeks, and concluded that, in trained animals, these muscles recovered total CSA to their pre‐injury values within 4 weeks and remained significantly greater than untrained SCI animals for up to 12 weeks.^17^ These results are consistent with our data and suggest that treadmill training accelerates recovery of muscle CSA.

Previous work has shown that complete SCI causes dramatic changes in fiber type composition in muscles below the injury toward fast phenotypes, especially in slow‐contracting muscles.^1^ Fiber type transformation has also been observed after incomplete SCI, but it is less marked.^3^ Both SOL and EDL muscles increase their expression of type IIX fibers in rats with incomplete SCI.^3^ The role of the type IIX fiber is not completely understood, but this fiber phenotype seems to be expressed in fibers undergoing transition from slow to fast twitch.^18^, ^19^ In our study, there was only a slight increase in fibers expressing type IIX fibers in the fast‐twitch muscles after SCI. There are several possible reasons. First, the relative proportion of type IIX fibers is known to vary considerably between species,^20^ which may explain the disparity between our findings in mice and those of Hutchinson *et al*.^3^ in rats. Second, it was sometimes difficult to distinguish type IIX and type IIB fibers, especially in the TA muscle. This would mean that some type IIX fibers were miscategorized. In contrast to the study by Glaser *et al*., where the mouse MG muscle was composed of some type I and IIA fibers, we only observed type IIB and IIX fibers.^21^ Thus, it is possible that some IIA fiber types were misclassified as type IIB or IIX. Another possibility is that the histochemistry protocol used in our study did not stain these fibers optimally; however, previous work using the same staining technique clearly differentiated the 3 classic fiber types (I, IIA, and IIB) in turtle neck muscle.^15^ It is notable that Glaser *et al*. stained the whole triceps surae muscle group (i.e., SOL, MG, and LG). In our experience, it has been very difficult to distinguish muscle borders. Thus, our approach avoids this possible confound. A sampling error is a less likely explanation, as both the deep and superficial parts of MG were assessed.

Treadmill training has been shown to have a limited effect on fiber type composition after SCI.^2^, ^13^, ^22^ None of the 3 training durations employed in our study significantly changed the fiber type composition of any muscle analyzed, even though 9 weeks of training decreased the extent of atrophy. This differential effect of treadmill training on muscle fiber size and fiber type composition has also been reported in normal mice. Glaser *et al*. showed that 6 weeks of treadmill training in normal uninjured mice increased muscle mass and capillary density but did not change the relative proportion of fiber types.^21^ Thus, any effect of exercise training on muscle fiber type composition may require a different type of stimulus or more intense exercise.

Muscle responses after SCI depend on the severity of the spinal injury and the specific muscles affected.^1^ Complete spinal cord transection leads to much greater muscle atrophy than incomplete injury, and studies in rats with complete SCI demonstrated that plantarflexor (generally more slow‐twitch) muscles are more affected than dorsiflexor (mostly fast‐twitch) muscles.^2^, ^23^ The effect of incomplete SCI on muscle properties is less clear. There is some evidence that a similar pattern of change occurs after incomplete SCI; that is, plantarflexor muscles exhibit greater muscle atrophy than dorsiflexor muscles.^17^ This differential response may be a result of the antigravity role of plantarflexor muscles; that is, they have higher neuromuscular activity than dorsiflexors.^24^ Therefore, physiological extensor muscles such as SOL exhibit greater atrophy when neuromuscular activity is diminished. In our study, the MG in the affected hindlimb of 10‐week untrained animals showed a greater degree of atrophy than the TA muscle when compared with control values. However, as the data show, the plantarflexor and slow‐contracting SOL muscle did not atrophy after injury. This finding may be due to a number of factors. First, hypertrophy in the SOL may be a compensation process related to atrophy in the MG. It is also possible that muscle atrophy decreases around the fourth week post‐injury when animals begin to show spontaneous recovery.^3^ In our study, atrophy in SOL may have occurred early after injury, but, by the fourth week post‐injury, the first time‐point for assessment, the atrophy had resolved. Mice have been shown to recover trunk stability earlier after injury (2 weeks) compared with rats (∼5 weeks), and this is mediated through weight bearing on the limbs, thereby leading to a lack of atrophy in the postural SOL muscle.^25^, ^26^ This may be particularly true in our study due to the hemisection lesion. Our study animals may have used their affected hindlimb to balance themselves more than animals with a spinal contusion, where both sides of the spinal cord, and thus both hindlimbs, are affected. Indeed, SOL was the only muscle that was significantly larger in the affected hindlimb compared with the non‐affected hindlimb of trained animals.

Treadmill training has been shown to ameliorate some of the muscle atrophy that occurs after SCI.^12^ However, there are scant data regarding the optimal treadmill training regimen after incomplete SCI. Our study has demonstrated that 9 weeks of treadmill training is more effective in restoring muscle CSA than 3 and 6 weeks of training. Nine weeks of training led to an increase in muscle fiber CSA only in the muscles that usually atrophy after SCI. The CSA of fast‐twitch muscles MG and TA was significantly larger in animals that underwent 9 weeks of training compared with untrained animals, whereas there was a limited effect on the slow‐twitch SOL muscle. Importantly, when compared with control (C10U) animals, the extent of MG muscle atrophy was less in animals that were trained for 9 weeks than in untrained animals (Table 4). Moreover, in muscle fibers that showed limited atrophy or hypertrophy after SCI, 9 weeks of training led to a larger CSA compared with the same muscles in control animals. Thus, 9 weeks of treadmill training ameliorates atrophy of muscles that are highly affected by SCI and leads to hypertrophy of muscles that are less affected or not affected by SCI.

Another finding from this study is that muscle fiber CSA in the affected hindlimb of animals trained for 3 weeks was smaller than in the affected hindlimb muscles of untrained animals. This suggests that treadmill training early after injury may have a detrimental effect on muscle properties. The loss of motor and sensory input after SCI leads to a period of extreme metabolic change and catabolism, and the additional stress associated with training early after injury may not be beneficial for muscle, or indeed the nervous system.^27^, ^28^, ^29^ It is noteworthy that initiating intensive exercise such as treadmill training so early after injury in humans is not feasible. Nevertheless, this is an important finding, and future clinical trials into the effectiveness of early exercise training after SCI may need to carefully consider the training dosage at this early stage.

The effect of other types of locomotor training to improve hindlimb muscle properties has also been investigated. In normal mice, voluntary wheel running for 4 weeks can lead to an increase in the expression of fast fiber types, especially in the TA muscle.^30^ After complete SCI, passive cycling for 1 week ameliorated muscle atrophy but had limited effects on fiber type composition.^31^ Similar to treadmill training, cycling exercise after SCI uses sensory feedback through rhythmic, alternating stretch of the right and left muscles to activate spinal cord circuits, which, in turn, influence the pattern‐generating circuits and motor neurons that innervate the hindlimb muscles. However, passive cycling after incomplete SCI was not shown to be superior to treadmill training in recovery of muscle size.^17^ Furthermore, this type of intervention was less effective than treadmill training in restoring TA muscle size. This has been attributed to the fact that the ankle is positioned in a dorsiflexed position during cycling, minimizing the stretch reflex in this muscle. In patients with SCI, there is some evidence that cycling combined with functional electrical stimulation can attenuate the loss of muscle mass.^32^, ^33^

Another component of the training that may also influence muscle recovery is treadmill speed. High treadmill speed (54 m/min) has been shown to increase activation of TA and MG muscles in normal rats.^34^ This could be beneficial to maintain muscle size after injury; however, due to the animal's disability, especially early after injury, high treadmill speeds may not be achievable. In our study, animals were trained at an average speed of 11 m/min, so it is possible that training at higher speeds would have led to significant muscle changes earlier than 10 weeks after injury. In‐cage activity may also influence muscle recovery and lead to a ceiling effect. The use of environmental enrichment, such as ladders and stairs, to add resistance to the muscle may supplement treadmill training and provide additional benefits to muscle recovery. In normal animals, ladder climbing with or without load attached to the tail, for example, leads to hindlimb muscle hypertrophy.^35^ In contused SCI rats, 8 weeks of staircase climbing training was also shown to improve MG and TA muscle mass.^36^ Interestingly, the addition of resistance to locomotor training by inclining the treadmill was demonstrated to only increase the activation of the MG muscle, and 6 weeks of uphill running (15°) did not significantly change whole muscle mass compared with flat treadmill running.^21^, ^34^ The effects of inclined treadmill training and resistance training on muscle properties after SCI, however, need further investigation.

In conclusion, 9 weeks of treadmill training after incomplete SCI in mice leads to an increase in fast‐twitch muscle fiber size, but has limited effects on slow‐twitch anti‐gravity muscles and muscle fiber type composition. Together, these data provide important evidence of the benefits of exercising partially paralyzed limbs after SCI and may also help improve activity‐based therapies for SCI patients.
